# Does the magnitude of injuries affect the outcome of proximal humerus fractures treated by locked plating (PHILOS)?

**DOI:** 10.1007/s00068-020-01451-9

**Published:** 2020-08-10

**Authors:** Till Berk, Sascha Halvachizadeh, Frederik Bellmann, Lucas Büsser, Hans-Christoph Pape, Florin Allemann

**Affiliations:** 1 Department of Trauma, University Hospital Zurich, Raemistrasse 100, 8091 Zurich, Switzerland; 2 Harald-Tscherne Laboratory for Orthopedic and Trauma Research, University of Zurich, Sternwartstrasse 14, 8091 Zurich, Switzerland

**Keywords:** PHILOS, DASH, Outcome after PHILOS, Proximal humerus fracture, ISS, Multiple injured, EQ-5D-3L, EQ-VAS

## Abstract

**Purpose:**

Health-related quality of life (HRQoL) becomes increasingly relevant in an aging society. Functional outcome (FO) and the patient-reported outcome (PRO) after surgical treatment of proximal humerus fractures (PHF) depends on numerous factors, including patient- and injury-specific factors. There is little evidence on how the FO and the PRO vary in different settings such as monotrauma or multiple injuries, even though the PHF is one of the more frequent fractures. In addition, to a previous study, on multiple injured patients and upper extremity injuries, the aim of the current study was to investigate the impact of multiple injuries, quantified by the ISS, on the FO and PRO after surgically treated PHF by PHILOS.

**Methods:**

A retrospective cohort-study was conducted with an additional follow-up by a questionnaire. HRQoL tools such as range of motion (ROM), the Quick-Disability of Arm Shoulder and Hand score (DASH), EuroQol Five Dimension Three Levels (EQ-5D-3L), and EuroQol VAS (EQ-VAS) were used. The study-population was stratified according to ISS obtained based on information at discharge into Group I/M-H (ISS < 16 points) and Group PT–H (ISS ≥ 16). Median outcome scores were calculated and presented. Inclusion criteria: adult patients (> 18 years) with PHF treated at one academic Level 1 trauma center between 2007 and 2017 with Proximal Humeral Inter-Locking System (PHILOS) and preoperative CT-scan. Group stratification according Injury Severity Score (ISS): Group PT–H (ISS ≥ 16 points) and Group I/M-H (ISS < 16 points). Exclusion criteria: oncology patients, genetic disorders affecting the musculoskeletal system, paralysis or inability to move upper extremity prior or after injury, additional ipsilateral upper limb fractures, open injuries, associated vascular injuries as well brachial plexus injuries and nerve damages. Follow-up 5–10 years including PRO: EQ-5D-3L and EQ-VAS. FO, including DASH and ROM. The ROM was measured 1 year after PHILOS.

**Results:**

Inclusion of 75 patients, mean age at injury was 49.9 (± 17.6) years. The average follow-up period in Group I/M-H was 6.18 years (± 3.5), and in Group PT–H 5.58 years (± 3.1). The ISS in the Group I/M-H was 6.89 (± 2.5) points, compared to 21.7 (± 5.3) points in Group PT–H (*p* ≤ 0.001). The DASH-score in Group I/M-H was 9.86 (± 13.12 and in Group PT–H 12.43 (± 15.51, n.s.). The EQ-VAS in Group I/M-H was 78.13 (± 19.77) points compared with 74.13 (± 19.43, n.s.) in Group PT–H. DASH, EQ-VAS as well as ROM were comparable in Groups I/M-H and PT–H (9.9 ± 13.1 versus 12.4 ± 15.5, n.s.). The EQ-5D-3L in Group I/M-H was 0.86 (± 0.23) points compared to Group PT–H 0.72 (± 0.26, *p* ≤ 0.017). No significant differences could be found in Group I/M-H and PT–H in the severity of traumatic brain injury (TBI). A multivariable regression analyses was performed for DASH, EQ-5D-3L and EQ-VAS. All three outcome metrics were correlated. There was a significant difference between the EQ-5D-3L and the ISS (Beta-Coefficient was 0.86, 95% low was 0.75, 95% high was 0.99, *p* ≤ 0.041). No significant correlation could be found comparing DASH, EQ-5D-3L and EQ-VAS to age, gender and TBIs.

**Conclusion:**

Multiple injuries did not affect the DASH, ROM or EQ-VAS after PHILOS; but a higher ISS negatively affected the EQ-5D-EL. While the ROM and DASH aim to be objective measurements of functionality, EQ-5D-3L and EQ-VAS represent the patients’ PRO. The FO and PRO outcomes are not substitutable, and both should be taken into consideration during follow-up visits of multiple injured patients. Future research should prospectively explore whether the findings of this study can be recreated using a larger study population and investigate if different FO and PRO parameters come to similar conclusions. The gained information could be used for an enhanced long-term evaluation of patients who suffered a PHF from multiple injuries to meet their multifarious conditions.

**Level of evidence:**

II.

## Introduction

PHFs are the second most common fractures of the upper extremities for the elderly, with constant increase in the number of patients suffering from PHFs [1, 2] and account for 5–6% of all fractures in the emergency department (incidence of 82 per 100,000 patients) [3, 4]. In severely injured patients (ISS ≥ 16), upper extremity fractures occurred in 21%, (9–23%) of cases [5, 6]. A fractured humerus required surgical treatment in 68.6% cases [5, 6]. PHILOS is the one of the favored bone-preserving surgical treatment for PHF [7, 8]. Several studies have investigated the FO after PHILOS treatment of PHF [2, 9, 10]. It has been shown that age (< 65 years), less complex fracture pattern, and isolated injuries resulted in improved functional outcome measurements [8]. PHFs have been found to reduce HRQoL from 0.86 (± 0.16) to about 0.65 (± 0.28) at 12 months follow-up [11]. A more recent study investigated the quality of life after open reduction internal fixation (ORIF) of PHF, and reported increasing age and more complex fracture types to be associated with a deceleration of functional improvement [9]. PRO are becoming progressively important when evaluating surgical treatments [12]. To date there are few studies that have investigated the HRQoL in multiple injured patients. A possible explanation could be that patient populations with a variety of possible complications, such as multiple injured patients provide, are often excluded from numerous existing PRO-studies [13–16]. This lack of information of PRO and multiple injured patients with orthopedic injuries has been previously described [17, 18].

In addition to a previous study on multiple injured patients and upper extremity injuries [19], the aim of this study was to investigate the impact of multiple injuries, quantified by the ISS, on the FO and PRO after surgically treated PHF by PHILOS.

## Methods

### Ethical consideration

This cohort-study study was approved by the Kantonale Ethik Kommission (KEK) Zurich and was performed under the BASEC-Nr. 2018–00,146 and BASEC Nr. 2018–00,842. Patients that were contacted during this study gave their verbal consent to participate. The study was conducted in accordance with the Declaration of Helsinki.

### Study population

### Inclusion criteria

This study included patients (ages 18 and older) that were treated between 2007 and 2017 at one academic Level 1 trauma center due to injury patterns including proximal humeral fractures. All included patients were treated with PHILOS. Only patients that had a preoperative CT-scan were included in this study. The PHF was classified using the Neer classification [20].

#### Exclusion criteria

Patients with genetic disorders affecting the musculoskeletal system, as well as patients with oncologic diseases, were excluded from this study. Patients with additional ipsilateral upper limb fracture, open injuries, associated vascular injuries as well brachial plexus injuries and nerve damages, were also excluded, along with patients requiring a long PHILOS plate.

#### Groups and definitions

The study population was stratified according to ISS obtained based on information at discharge [21]: Group I/M-H (ISS < 16 points) and Group PT–H (ISS ≥ 16). The surgical technique as well as the postoperative procedure has previously been described [22, 23]. All patients were treated according to the in-hospital guidelines: Antero-lateral approach to the shoulder joint with open reduction and internal fixation using either a 3- or 5-hole PHILOS plate. All patients had early postoperative functional treatment including physiotherapeutic-assisted movement. Elevation above 90° was allowed after 6 weeks, and weight bearing after 12 weeks after surgery. Clinical and radiological follow-ups were performed after 6 and 12 weeks, and 6 and 12 months. Patients that did not adhere to this treatment plan were excluded from this study.

#### Outcome

**Fig. 1:**
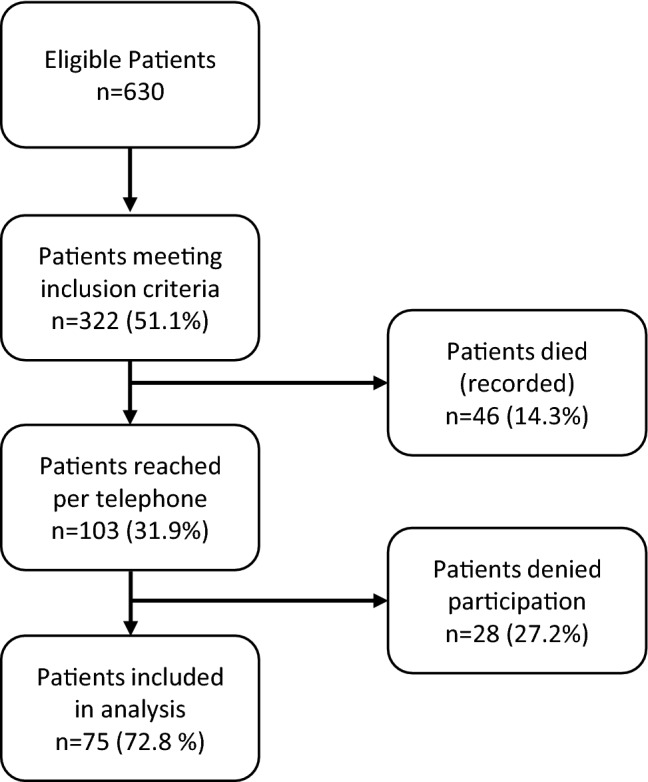
Flow Chart of included patients

For FO the ROM was used, which was measured during routine follow-up visit 1 year after injury. The ROM was quantified, with a hand-held goniometer, rounded up to the nearest 5°. A follow-up questionnaire was used, to obtain additional data regarding the study population which has previously been described. To initiate post-operative contact, patients were telephoned, utilizing the phone numbers found in our information system. The follow-up questionnaires were performed in May and June 2019. Questionnaires were completed after a median of 6.2 years since injury. The response rate was 72.8%. An overview of the excluded patients is shown as a flow chart in Fig. 1.

HRQoL was assessed, using the German EQ-5D-3L and the EQ-VAS. The EQ-5D-3L is described as a standardized instrument for measuring the general health status. It is established on the level of suffered problems (no problems, some problems, extreme problems) for five dimensions (self-care, usual activities, mobility, pain or discomfort, and depression or anxiety) [24, 25]. The scores were calculated by using a scoring algorithm appropriate for a population of Swiss patients [26], with a possible range from − 0.33 to 1.00. The EQ-VAS was developed to document an individual’s current self-rated health on a scale from 0 to 100 [27]. The higher the score for both scores, the higher the HRQoL [24]. Further PRO was assessed using a German version of the quick DASH score [28].

#### Traumatic brain injury

TBI was defined according to Glasgow Coma Scale (GCS) at admission [29]. Based on GCS, patients were stratified according to severity of TBI following ATLS-Guidelines [30].

### Statistical analysis

Continuous variables are summarized as mean with standard deviation (± SD). Categorical variables are displayed as count and percentages. Two groups on continuous variables were compared using the students’ *t* test; for groups on binary variables the chi-square test was used. ANOVA was used when comparing more than two groups. A *p* value below 0.05 was considered statistically significant. The effect of “multiple injured” as defined by ISS ≥ 16 points on the outcome parameter EQ-5D-3L, EQ-VAS, and DASH were analyzed using multivariable linear mixed model regression analysis. Variables included in this model were gender, age at injury, and traumatic brain injury, based on clinical and statistical relevant impact on the outcome parameter. All analyses have been performed using R (R Core Team 2019). R: A language and environment for statistical computing. R Foundation for Statistical Computing, Vienna, Austria. URL https://www.R-project.org/). All inserted tables were created with Microsoft PowerPoint Version 2016.

## Results

### Study population

This study included 75 patients, mean age at injury of 49.92 years (± 17.56 years), and 42 (56.0%) females. The mean follow-up was 5.94 years (± 3.33 years). PHFs were two segmented in 14 (18.7%) of cases, three segmented in 38 (50.7%), and four-segmented in 23 (30.7%) cases. The demographics of included patients are summarized in Table 1.

**Table 1:** Patient demographics

*n*	75
Age at injury, years (mean (SD))	49.92 (± 17.56)
Female gender [*n* (%)]	42 (56.0)
LOS, days [mean (SD)]	13.40 (± 12.89)
Multiple injured (ISS ≥ 16 points) [*n* (%)]	30 (40.0)
ISS, points [mean (SD)]	12.83 (± 8.26)
AIS extremity, points [mean (SD)]	2.72 (± 0.71)
TBI [*n* (%)]	
1° (Mild)	60 (80.0)
2° (Moderate)	6 (8.0)
3° (Severe)	9 (12.0)
Neer [*n* (%)]	
2	14 (18.7)
3	38 (50.7)
4	23 (30.7)
Follow-up time, years (mean (SD))	5.94 (± 3.33)

*Tbi* traumatic brain injury, *n* number, *SD* standard deviation, *n.s.*  not significant (*p* > 0.05), *LOS *length of stay in days, *AIS* abbreviated injury scale, *ISS* injury severity score

### Impact of multiple injuries on functional outcome and quality of life

Group I/M-H (ISS < 16 points) included 45 (60%) and Group PT–H (ISS ≥ 16 points) 30 (40%) patients. The mean follow-up in Group I/M-H was 6.2 years (± 3.47) vs. Group PT–H 5.6 years (± 3.14, n.s.). Group PT–H was significantly younger compared with Group I/M-H (42.7 ± 15.3 years versus 54.7 ± 17.5 years, p ≤ 0.003). Patients in Group PT–H were hospitalized significantly longer (21.8 ± 15.9 days) compared with Group I/M-H (7.8 ± 5.7, *p* ≤ 0.001). The mean ISS of Group PT–H was 21.7 (± 5.26,) compared with 6.9 ± 2.5 points in Group I/M-H and, therefore, significantly higher (*p* ≤ 0.001). No significant differences could be found in Group I/M-H and PT–H in the severity of the TBI. (Table 2).

**Table 2:** Injury distribution

	Group I/M-H	Group PT–H	*p* value
*n*	45	30	
Age at injury, years [mean (SD)]	54.73 (± 17.46)	42.72 (± 15.33)	0.003
Female gender [*n* (%)]	34 (± 75.6)	8 (± 26.7)	< 0.001
LOS, days [mean (SD)]	7.82 (± 5.68)	21.77 (± 15.95)	< 0.001
ISS, points [mean (SD)]	6.89 (± 2.50)	21.73 (± 5.26)	< 0.001
AIS extremity, points [mean (SD)]	2.58 (± 0.50)	2.93 (± 0.91)	n.s
TBI [*n* (%)]			n.s
1° (Mild)	41 (91.1)	19 (63.3)	
2° (Moderate)	1 ( 2.2)	5 (16.7)	
3° (Severe)	3 ( 6.7)	6 (20.0)	
Neer [*n* (%)]			n.s
2	9 (20.0)	5 ( 16.7)	
3	23 (51.1)	15 ( 50.0)	
4	13 (28.9)	10 ( 33.3)	

*Tbi* traumatic brain injury, *n.s.*  not significant (*p* > 0.05), *SD* standard deviation, *n* number, *LOS *length of stay in days, *AIS* Abbreviated Injury Scale, *ISS* Injury Severity Score

### Outcome parameter

The EQ-5D-3L was significantly lower in Group PT–H (0.72 ± 0.26, *p* ≤ 0.017) compared with Group I/M-H (0.86 ± 0.23). No significant differences between Group I/M-H and Group PT–H could be found in DASH and EQ-VAS (Table 3). The ROM did not differ comparing Group I/M-H with PT–H (Table 4).

**Table 3:** Outcome parameter

	Group I/M-H	Group PT–H	*p* value
*n*	45	30	
Age at questioning, years [mean (SD)]	60.91 (± 16.80)	48.30 (± 15.13)	0.001
Follow-up, years [mean (SD)]	6.18 (± 3.47)	5.58 (± 3.14)	n.s
DASH-Score [mean (SD)]	9.86 (± 13.12)	12.43 (± 15.51)	n.s
EQ-5D-3L [mean (SD)]	0.86 (± 0.23)	0.72. (± 0.26)	0.017
EQ-VAS [mean (SD)]	78.13 (± 19.77)	74.13 (± 19.43)	n.s

*n.s.*  not significant (*p* > 0.05), *n* number, *SD* standard deviation, *DASH* disability of the arm, shoulder and hand

**Table 4:** Range of motion after 12 months in angular degrees

	Group I/M-H	Group PT–H	
*n*	45	30	
Retroversion [mean (SD)]	29.78 (± 11.94)	39.44 (± 6.98)	n.s
Flexion [mean (SD)]	133.06 (± 29.86)	145.37 (± 42.99)	n.s
Abduction [mean (SD)]	119.72 (± 35.00)	142.41 (± 42.46)	n.s
Adduction [mean (SD)]	43.28 (± 12.24)	38.30 (± 7.66)	n.s
External Rotation [mean (SD)]	59.17 (± 16.65)	61.11 (± 19.43)	n.s
Internal Rotation [mean (SD)]	73.53 (± 16.56)	74.81 (± 19.68)	n.s

*n* number, *SD* standard deviation, *n.s.* not significant (*p* > 0.05)

A multivariable regression analyses were performed for DASH, EQ-5D-3L, and EQ-VAS. All three-outcome metrics were correlated. There was a significant difference for the EQ-5D-3L and the ISS (Beta-Coefficient was 0.86, 95% low was 0.75, 95% high was 0.99, *p* ≤ 0.041). No significant correlation could be found comparing DASH, EQ-5D-3L, and EQ-VAS to age, gender, and TBI’s. Therefore, TBI did not affect the DASH, the EQ-5D-3L or the EQ-VAS within the groups. (Table 5).

**Table 5:** Multivariable regression analysis of EQ-5D-3L, EQ-VAS and DASH

	Co-variables	Beta-coefficient	95% CI	*p* value
EQ-5D-3L	ISS > 16	0.86	0.75–0.99	0.041
	Sex (female)	0.95	0.83–1.10	n.s
	Age at injury	1.00	1.00–1.01	n.s
	Minor TBI	Reference		
	Moderate TBI	1.00	0.80–1.24	n.s
	Severe TBI	1.03	0.86–1.24	n.s
EQ-VAS	ISS > 16	− 5.63	5.89 to − 0.96	n.s
	SEX (female)	1.28	5.70–0.22	n.s
	Age at injury	− 0.09	0.15 to − 0.59	n.s
	Minor TBI	Reference		
	Moderate TBI	7.69	9.05–0.85	n.s
	Severe TBI	0.61	7.44–0.08	n.s
DASH	ISS > 16	5.66	4.18–1.36	n.s
	SEX (female)	2.73	4.04–0.68	n.s
	Age at injury	0.14	0.11–1.311	n.s
	Minor TBI	Reference		
	Moderate TBI	0.65	6.41–0.10	n.s
	Severe TBI	− 1.42	5.27 to − 0.27	n.s

*Tbi* traumatic brain injury, *n.s.*  not significant (*p* > 0.05), *ISS* injury severity score, *DASH* disability of the arm, shoulder and hand

## Discussion

This study investigated the HRQoL based on FO, DASH, ROM, as well as PRO, EQ-5D-3L, and EQ-VAS, comparing multiple injured patients stratified according to ISS in Group PT–H (ISS ≥ 16) with Group I/M-H (ISS < 16 points). The following points can be concluded:

1. The FO, DASH, and ROM were comparable in Group I/M-H and Group PT–H.
2. Group I/M-H and Group PT–H had a comparable EQ-VAS after PHILOS.
3. Despite no significant differences in EQ-VAS, PRO (EQ-5D-3L) was significantly lower in Group PT–H, compared with Group I/M-H.

### Functional outcome

Previous studies have shown that injury pattern in patients sustaining a polytrauma affects mortality, physical outcome, and posttraumatc quality of life [5, 31]. Contrary to this study’s results, Banerjee et al. showed that polytraumatized patients with upper extremity injuries have often persistent severe functional restrictions and will not regain their pre-injury level of function [5]. Yet, it has also been shown that injuries to the uppr extremity are not predictive for the development of poor clinical outcomes in multiple injured patients [32] (see Table 6).

**Table 6:** Subgroup Analysis

	Group I/M-H				Group PT–H			
	Mild	Moderate	Severe	*p*	Mild	Moderate	Severe	*p*
*n*	41	1	3		19	5	6	
Age at injury, years [mean (SD)]	53.56 (17.41)	52.73 (NA)	71.38 (14.20)	NA	44.09 (14.23)	42.83 (22.59)	38.28 (13.99)	n.s
Female gender [*n* (%)]	31 ( 75.6)	1 (100.0)	2 ( 66.7)	n.s	3 ( 15.8)	2 ( 40.0)	3 ( 50.0)	n.s
LOS, days [mean (SD)]	6.80 (4.41)	10.00 (NA)	21.00 (6.08)	NA	21.37 (17.86)	21.40 (12.16)	23.33 (14.36)	n.s
Neer [*n* (%)]				n.s				n.s
2	8 ( 19.5)	0 ( 0.0)	1 ( 33.3)		5 ( 26.3)	0 ( 0.0)	0 ( 0.0)	
3	23 ( 56.1)	0 ( 0.0)	0 ( 0.0)		10 ( 52.6)	2 ( 40.0)	3 ( 50.0)	
4	10 ( 24.4)	1 (100.0)	2 ( 66.7)		4 ( 21.1)	3 ( 60.0)	3 ( 50.0)	
ISS, points [mean (SD)]	6.93 (2.49)	4.00 (NA)	7.33 (2.89)	NA	21.37 (4.83)	22.00 (5.79)	22.67 (6.95)	n.s
Follow-up time, years [mean (SD)]	6.13 (3.42)	10.27 (NA)	5.62 (4.74)	NA	5.38 (3.06)	6.57 (3.62)	5.39 (3.44)	n.s
EQ-VAS [mean (SD)]	78.44 (19.88)	90.00 (NA)	70.00 (22.91)	NA	71.58 (22.11)	79.80 (20.68)	77.50 (4.18)	n.s
DASH [mean (SD)]	9.80 (13.34)	0.83 (NA)	13.74 (12.69)	NA	12.46 (16.55)	15.67 (19.94)	9.63 (8.39)	n.s
EQ-VAS [mean (SD)]	0.85 (0.23)	1.00 (NA)	0.92 (0.14)	NA	0.73 (0.28)	0.67 (0.30)	0.73 (0.22)	n.s

Frima et al. has further been shown that increased age, less complex fracture pattern, and isolated injuries result in improved functional outcomes after PHF [8]. Similar findings could not be observed in any of this study’s results. Although Group I/M-H was significantly older than Group PT–H, no difference in FO was observed. A possible explanation for this could be the relatively small age difference with a rather high standard deviation.

There was also no significant differences found in fracture morphology in this study, since the Neer classification did not show any significant differences. Furthermore, a very reliable classification can be assumed, since all patients received a CT of the PHF preoperatively. though more severe PHF with local concomitant injury were excluded from the study. This could also represent a limitation in the statement regarding the FO and, therefore, might not be comparable. Since Group PT–H was significantly younger compared with Group I/M-H, age might affect the functional outcome as well. It could be argued that younger patients (Group PT–H), despite a significant higher ISS, have a better outcome than older patients (Group I/M-H) [33]. On the other hand, it could be assumed that younger patients often have a higher demand on their HRQoL and that this demand is lower in older patients, especially if one takes into consideration that Group I/M-H was shortly before their retirement in our population, when conducting the questioner.

Previous studies have shown that the ROM after upper extremity fracture depends on fracture location and associated injuries [19]. However, according to the findings in this study, the ROM with comparable fracture location and patterns seems to be independent from additional injuries. Further it has been shown, that those ROM are depending on treatment strategy [34]. The current study investigated only fractures that were treated with the same strategy (PHILOS). This could be a possible limitation. To make a stronger statement about the FO, a larger cohort size would be necessary.

### Patient-reported outcome

The current study revealed a discrepancy of stated subjective general health status and the calculated EQ-5D-3L score when comparing Group I/M-H with Group PT–H. McPherson et al. showed that populations EQ-VAS ratings of conditions can vary, especially when the condition is more severe [35]. This precious finding may have influenced our Group PT-U and may, therefore, have led to the lack of significance, although the multivariable regression analyses could not confirm a correlation between EQ-VAS and ISS, age, gender or TBI.

Contrary to the results of this study, van der Vliet et al. [36] investigated the impact of injury severity and injury distribution on the outcome of upper extremity fracture and found that the EQ-5D is not affected by polytrauma in extraarticular fractures. Furthermore, they showed that the quick DASH is affected by increasing trauma energy [36]. Since only intraarticular PHF were analyzed in this cohort, the results might not be comparable.

The current data indicate that the EQ-5D-3L is significantly influenced by the ISS and seems to be independent of age, sex, and TBIs. However, this result could not be observed on the FO. Only at the long-term follow-up in PRO, this difference became apparent. Therefore, it might be helpful in future studies to include additional FO parameters and a variety of PRO parameters in order to obtain a better assessment of the HRQoL of multiple injured patients.

The TBI data were included in this study in order to minimize a potential impact of structural brain injury on FO and PRO. Since no significant differences between the groups could be found and even the multivariable regression analyses could not show any correlation, it is assumed that the Group I/M-H and Group PT–H are well comparable and that this could be a strength of this study.

### Limitations

There are certain limitations of this study. Important differences could be identified in demographic characteristics between the groups: Group PT–H is older compared to Group I/M-H. This might influence the FO and PRO. Nevertheless, the majority of patients per group were in working ages indicating similar functional requirements. Since this was a retrospective study, not all potential influencing factors could have been addressed.

Further, the number of participants per group might indicate a type two error. This is based on the response rate. However, during the interview patients were able to directly ask and clarify questions that might not be understood in a paper-based questionnaire. This increases confidence of the correctness of answers. Furthermore, this study represents a retrospective analysis and only associations are reported that do not necessarily represent a larger population.

Another limitation could be that the questionnaires were conducted at different lengths of follow-ups. It has been previously reported that outcome scores can change over time and that they can reach a plateau, after 1 year [37, 38]. This could affect the HRQoL after trauma, depending on the follow-up periods. The ROM was measured 1 year after the surgery and the questionnaires in the present study were carried out after a median of 6.2 years since injury.

Finally, the major disadvantage of the DASH and ROM are that they might not allow a comparison of the outcome in patients with different or multiple injuries, which has been previously reported [39]. The DASH reflects the function of the entire upper arm and is not specific to PHF. Still, it is believed that multiple examinations of the same symptomatology improves the scientific quality of statements. To a certain extent, to overcome this disadvantage, instruments for assessing the HRQoL have been additionally used in this study.

## Conclusion

Multiple injuries did not affect the DASH, ROM or EQ-VAS after PHILOS, but a higher ISS negatively affected the EQ-5D-EL. While the ROM and DASH aim to be objective measurements of functionality, EQ-5D-3L and EQ-VAS represent the patients PRO. These outcomes are not substitutable, and both the FO and PRO should be taken into consideration during follow-up visits of multiple injured patients. Future research should prospectively explore whether our findings can be recreated with a larger study population and if different FO and PRO parameters come to similar conclusions.

The gained information could be used for an enhanced long-term evaluation of patients who suffered a PHF from multiple injuries to meet their multifarious conditions.
